# Validation of Polymorphisms Associated with the Risk of Radiation-Induced Oesophagitis in an Independent Cohort of Non-Small-Cell Lung Cancer Patients

**DOI:** 10.3390/cancers13061447

**Published:** 2021-03-22

**Authors:** Miguel E. Aguado-Barrera, Laura Martínez-Calvo, Juan Fernández-Tajes, Patricia Calvo-Crespo, Begoña Taboada-Valladares, Ramón Lobato-Busto, Antonio Gómez-Caamaño, Ana Vega

**Affiliations:** 1 Grupo Genética en Cáncer y Enfermedades Raras, Instituto de Investigación Sanitaria de Santiago de Compostela (IDIS), Fundación Pública Galega de Medicina Xenómica (FPGMX), 15706 Santiago de Compostela, A Coruña, Spain; miguelelias.aguado@usc.es (M.E.A.-B.); laura.martinez.calvo@usc.es (L.M.-C.); juan.fernandez.tajes@usc.es (J.F.-T.); 2 Department of Radiation Oncology Hospital Clínico Universitario de Santiago de Compostela, Servizo Galego de Saúde (SERGAS), Grupo Genética en Cáncer y Enfermedades Raras, Instituto de Investigación Sanitaria de Santiago de Compostela (IDIS), 15706 Santiago de Compostela, A Coruña, Spain; patricia.calvo.crespo@sergas.es (P.C.-C.); maria.begona.taboada.valladares@sergas.es (B.T.-V.); 3 Department of Medical Physics Hospital Clínico Universitario de Santiago de Compostela Servizo Galego de Saúde (SERGAS), 15706 Santiago de Compostela, A Coruña, Spain; ramon.lobato.busto@sergas.es; 4 Department of Radiation Oncology, Hospital Clínico Universitario de Santiago de Compostela, Servizo Galego de Saúde (SERGAS), 15706 Santiago de Compostela, A Coruña, Spain; antonio.gomez.caamano@sergas.es; 5 Grupo Genética en Cáncer y Enfermedades Raras, Instituto de Investigación Sanitaria de Santiago de Compostela (IDIS), Fundación Pública Galega de Medicina Xenómica (FPGMX), Biomedical Network on Rare Diseases (CIBERER), 15706 Santiago de Compostela, A Coruña, Spain

**Keywords:** radiotherapy, non-small-cell lung cancer, radiation adverse effects, radiation-induced oesophagitis, SNPs, validation, meta-analysis

## Abstract

**Simple Summary:**

Genetic variants identified in association with radiation therapy side effects in non-small-cell lung cancer patients require an independent validation. Therefore, the aim of our study was to replicate, in an independent cohort, the analyses of previously published studies associating single-nucleotide polymorphisms with radiation-induced oesophagitis. Following the original models, 2 of the 18 variants associated with radiation-induced oesophagitis in non-small-cell lung cancer patients were confirmed. Furthermore, we meta-analysed our cohort together with those of the reference studies. Twelve variants located in genes of inflammation and DNA double-strand break repair pathways remained associated with oesophagitis. These variants could be included in models for clinical prediction of radiation-induced oesophagitis to evaluate their performance.

**Abstract:**

Several studies have identified single-nucleotide polymorphisms (SNPs) associated with adverse effects in non-small-cell lung cancer (NSCLC) patients treated with radiation therapy. Here, using an independent cohort, we aimed to validate the reported associations. We selected 23 SNPs in 17 genes previously associated with radiation-induced oesophagitis for validation in a cohort of 178 Spanish NSCLC patients. Of them, 18 SNPs were finally analysed, following the methods described in the original published studies. Two SNPs replicated their association with radiation-induced oesophagitis (rs7165790 located in the *BLM* gene: odds ratio (OR) = 0.16, 95% CI = 0.04–0.65, *p*-value = 0.010; rs4772468 at *FGF14*: OR = 4.36, 95% CI = 1.15–16.46, *p*-value = 0.029). The SNP rs2868371 at *HSPB1* was also validated but displayed an opposite effect to the formerly described (OR = 3.72; 95% CI = 1.49–9.25; *p*-value = 0.004). Additionally, we tested a meta-analytic approach including our results and the previous datasets reported in the referenced publications. Twelve SNPs (including the two previously validated) retained their statistically significant association with radiation-induced oesophagitis. This study strengthens the role of inflammation and DNA double-strand break repair pathways in the risk prediction of developing radiation-induced oesophagitis in NSCLC patients. The validated variants are good candidates to be evaluated in risk prediction models for patient stratification based on their radiation susceptibility.

## 1. Introduction

Radiotherapy (RT) is one of the most important therapeutic modalities for patients with locally advanced non-small-cell lung cancer (NSCLC) [1,2,3,4]. The optimal dose and the success of RT is often limited by the tolerance of the surrounding normal tissue [5]. The oesophagus is one of the common organs that develops radiation-induced toxicity in patients with thoracic RT. The clinical manifestation resulting from inflammation, oedema, erythema or erosion of the oesophageal mucosa is oesophagitis [6,7]. It usually occurs 2 or 3 weeks after the beginning of treatment, and it can be present for months. Severe oesophagitis may even require more invasive medical or surgical interventions, which could negatively affect the patient’s quality of life [7].

Current radiation treatment regimens are designed to reduce the population-level risk of severe effects; however, some patients are more sensitive than others despite having similar clinical features (e.g., clinical stage, additional treatments or comorbidities). Part of this variation in the development of adverse effects is attributed to genetics. Radiogenomics studies the possible link between genetic variation and normal tissue toxicity after RT [8], with the goal of identifying genetic markers to include in predictive models. This would allow the stratification of patients based on the risk of adverse effects prior to treatment.

Studies investigating the role of genetic variants and radiation-induced oesophagitis in lung cancer have been carried out using a candidate gene approach, particularly focused on single-nucleotide polymorphisms (SNPs) in genes associated with stress response. These genes have been mainly linked with heat-shock proteins [9], inflammation [10,11,12] and DNA double-strand break (DSB) repair pathways [13]. Still, multiple treatment modalities (different RT techniques; RT alone or combined with chemotherapy), the variability in toxicity assessment scales and reduced cohort sizes have resulted in poor validation rates in independent cohorts. So far, only the SNP in the *HSPB1* gene has been reassessed in association with oesophagitis [9,14] and pneumonitis [15,16].

In this validation study, we aimed to replicate previously validated genetic associations with radiation-induced oesophagitis in an independent cohort of 178 Spanish NSCLC patients receiving radio(chemo)therapy. In view of the constraints encountered, and seeking to maximise statistical power [17], we alternatively applied a meta-analytic approach including our cohort as a second validation set.

## 2. Materials and Methods

### 2.1. Study Population and Data Collection

One hundred and seventy-eight patients with diagnosis of stages I–III NSCLC who completed the 3-month follow-up were selected from the RADIOGEN-Lung cohort (a study on the genomic influence in patients undergoing RT) that comprises 514 Galician individuals (northwest Spain). Patients were recruited at the Radiation Oncology Department of the Clinical University Hospital of Santiago de Compostela, Spain, between March 2008 and May 2015. Written informed consent was obtained from each subject according to the protocols approved by the ethics review board of the Galician Ethical Committee for Clinical Research and in compliance with the Declaration of Helsinki.

The patients were followed by the radiation oncologist according to the institutional follow-up guidelines: a visit prior to the start of RT and one first follow-up at 4 weeks post-RT, with thoracic-abdominal computed tomography (CT) screening. From 3 months to the first 3 years post-RT, the patient continues with a follow-up every 3 months and a tomographic follow-up every 3 or 6 months, depending on the symptoms. Next, the patient continues with check-ups every 6 months, with an annual tomography scan for the next 2 years. In addition, any medical assistance received for oesophageal symptoms that appear in the medical records is recorded.

Dosimetry, epidemiology, pathology and clinical and longitudinal side-effect data were collected for each patient using a case report form. Oesophagitis-related signs and symptoms, as well as their management and treatments, were collected from medical records. The severity of toxicities was graded by physicians according to the National Cancer Institute Common Terminology Criteria for Adverse Events (CTCAE) guidelines (version 4.03).

### 2.2. Radiation Treatment Characteristics

Patients underwent RT and chemotherapy (concomitantly or sequentially) or definitive RT, with or without induction chemotherapy. Medical oncologists administered cisplatin chemotherapy according to guidelines. All patients were treated with curative intent, using the three-dimensional conformal radiation therapy (3D-CRT). RT was delivered with a linear accelerator using 6 or 15 MV photon beams. The target dose was 60–66 Gy in 30–37 fractions at 1.8 to 2 Gy per fraction.

The treatment position was verified and corrected using a regular CT simulator, with the patient in a supine position with both arms above the head. CT slices (3 mm thick) were obtained starting at the level of the cricoid cartilage and including the entire volume of the liver, using intravenous contrast unless medically contraindicated. Gross tumour volume (GTV) delineation was performed, encompassing all known tumours identified by radiologic imaging (on the CT or positron emission tomography/computed tomography (PET/CT) scan using appropriate window-level settings) or bronchoscopy. Involved lymph nodes were delineated on the CT in the mediastinum. Fluorodeoxyglucose–positron emission tomography (FDG-PET)-negative nodes were not included unless pathologically proven malignant. Non-elective mediastinum irradiation was carried out. The clinical target volume (CTV) is defined as the GTV plus a margin of 0.5–1.0 cm. Generally, the CTV is extended 1 cm in all directions to create the planning tumour volume (PTV) and 1.5 cm up or down in the case of tumours located in the lower lobe with important displacements during breathing.

The planification was done with a fusion of CT and linear accelerator (FOCAL) unit, and planning was performed on a XiO treatment planning system using a superposition algorithm considering inhomogeneity corrections, according to the International Commission on Radiation Units and Measurements (ICRU) Report 50. Quantitative dose–volume analyses were performed using cumulative dose–volume histograms (DVHs) for the organs at risk (OARs), including the lung, oesophagus, heart and spinal cord. Dose distributions and DVHs of the OARs were calculated from the 3D-CRT treatment plans. For each patient, the oesophagus was delineated from the inferior border of the cricoid cartilage to the gastro-oesophageal junction, including from the entire thickness of the wall to the adventitia.

### 2.3. SNP Selection

A PubMed search of studies regarding radiation-induced oesophagitis of RT in NSCLC patients was conducted in December 2019 with the following terms: (((radiation[Title/Abstract]) OR (radiotherapy[Title/Abstract])) AND ((polymorphisms[Title/Abstract]) OR (variants[Title/Abstract]) OR (variant[Title/Abstract) OR (SNP[Title/Abstract)) AND ((oesophagitis[Title/Abstract]) OR (oesophageal[Title/Abstract])) AND ((non-small-cell lung cancer[Title/Abstract]) OR (lung cancer[Title/Abstract]))).

Thirteen publications were identified. Only studies with SNP–phenotype associations confirmed in validation cohorts were selected: López-Guerra et al. [9,10], Pu et al. [11] and Zhao et al. [13]. These studies included genes linked to heat-shock proteins [9], inflammation [10,11] and DNA DSB repair pathway genes [13]. Altogether, the selected studies reported 23 unique validated SNPs with significant associations with oesophagitis (Table S1).

### 2.4. DNA Extraction and Genotyping

Genomic DNA was extracted according to manufacturer recommendations from peripheral blood (collected at the visit prior to the start of RT) with the automatic Chemagen Robot (Chemagen Biopolymer-Technolgie AG, Beasweiler, Germany) at the Fundación Pública Galega de Medicina Xenómica (FPGMX) in Santiago de Compostela, Spain. Genotyping was conducted with the Agena Bioscience MassARRRAY^®^ technology; PCR primers for multiplex assays were designed using Assay Design Suite v2.0 software (Agena Bioscience^®^. San Diego, CA, USA). The SNPs rs3819721 (*TAP1*) and rs204993 (*AGER*) were excluded due to genotyping design reasons. Genotype calling was performed with MassARRAY® Typer v4.0 software (Agena Bioscience^®^. San Diego, CA, USA) by pooling all the study data simultaneously. Only SNPs with a genotyping call rate of over 95% were included in the study.

Twenty-one SNPs related to oesophagitis passed quality control and were in Hardy–Weinberg equilibrium. The very low minor allele frequency of three SNPs located in the *BRCA1* gene (rs12516, rs1799966, rs8176257) made it impossible to investigate them as described in the reference study (recessive genetic model).

### 2.5. Statistical Analysis

The statistical analyses used for replication mimic those described in the original studies. In short, for Pu et al. [11] and Zhao et al. [13], multivariate logistic regression was carried out to assess the influence of each SNP (using the inheritance model described in the reference study) on the risk of acute oesophagitis (following the definition criteria used by each of the reference studies; Table S2).

To validate the results from López Guerra et al. [9,10], Cox proportional hazard analysis was performed to calculate the hazard ratio (HR) and confidence interval (CI) of each genotype on toxicity endpoint risk. Patients without any adverse effect were censored at 15 weeks after starting RT, the date of last follow-up or death. Multivariate Cox regression analysis was carried out to adjust for covariates. Kaplan–Meier analyses were used to estimate the cumulative risk of radiation-induced oesophageal toxicity probability.

For individual SNP analysis, the Benjamini–Hochberg false discovery rate (FDR) multiple testing correction was applied. In all cases, a two-sided *p*-value of < 0.05 was considered statistically significant.

Apart from the replication analysis, a meta-analysis approach was also applied for the Pu et al. [11] and Zhao et al. [13] SNPs, joining our results to the previous original discovery and validation summary data. This was encouraged by an a priori meta-analysis power calculation [18] considering the following: (i) expected effect size (odds ratio (OR)) of 2, (ii) an incidence of the adverse effect of 20%, (iii) an average cohort size (*n* = 190) and (iv) an alpha level of 0.05. Even with moderate heterogeneity among the three cohorts, the power estimation would be above 80%. To decide whether the fixed effects or the random effects model should be applied in the meta-analysis, a Cochran Q statistic heterogeneity test was performed. Due to the small number of studies available (*n* = 3 cohorts), a conservative alpha value was set (alpha = 0.1).

All the analyses were performed in R software [19], using the packages rms [20], survival [21,22] and meta [23]. Our results were reported considering the STROGAR guidelines [24].

## 3. Results

### 3.1. Patient Characteristics

Table 1 summarises the patient and treatment characteristics of the validation cohort. We noted the low presence of radiation-induced oesophagitis and a preserved respiratory function in most patients. Even when they were smokers, they had a lower consumption of tobacco products per day compared with the cohorts in the original papers. In the dosimetry, all RADIOGEN-Lung patients were treated with the same RT technique (3D-CRT).

We were able to analyse 16 SNPs in a final validation cohort size of 113 patients from Pu et al. [11] and Zhao et al. [13] and 2 SNPs from López-Guerra et al. [9,10], with a final validation cohort size of 162 patients.

### 3.2. Replication Analysis

Two SNPs were replicated in our cohort: rs4772468, located in the *FGF14* gene and associated with an increased risk of oesophagitis at 3 months (OR = 4.36; 95% CI = 1.15–16.46; *p*-value = 0.029), and rs7165790, located in the *BLM* gene and previously found to have a protective effect for oesophagitis (OR = 0.16; 95% CI = 0.04–0.65; *p*-value = 0.010). However, these associations were not significant after FDR multiple testing correction (Table 2).

We found the SNP rs2868371 in *HSPB1* associated with oesophagitis CTCAE grade of ≥2 at the 15-week follow-up (HR = 3.72; 95% CI = 1.49–9.25; unadjusted *p*-value = 0.004; adjusted *p*-value = 0.083) (Table 2). Cox regression and Kaplan–Meier results showed that the CG/GG genotype was related to an increased risk of developing oesophagitis (*p*-value = 0.004; Figure S1A). Patients harbouring the rs2868371 CG/GG genotype presented a higher incidence of oesophagitis compared with patients with the CC genotype. This difference remains when considering chemoradiation (concurrent chemoradiation, *p*-value = 0.009; Figure S1B), dosimetric parameters, such as the mean oesophagus dose (≤31 Gy, *p*-value = 0.024; Figure S1C) and the percentage of oesophagus volume treated to ≥40 Gy (V40 ≤ 44%, *p*-value = 0.031; V40 > 44%, *p*-value = 0.035; Figure S1D).

The SNP rs1800469 in *TGFB1* described in [10] was not associated with the risk of oesophagitis in our cohort (HR = 0.67; 95% CI = 0.28–1.63; unadjusted *p*-value = 0.385; Figure S2).

### 3.3. Meta-Analysis Approach

Twelve out of the sixteen meta-analysed SNPs preserved their statistically significant association with radiation-induced oesophagitis (Table 2). To include our cohort as an additional validation set refined the previously reported associations, slightly enhancing the effect size for six SNPs: two of the SNPs from Pu et al. [11] (rs4772468 in *FGF14* and rs270771 in *LILRP2*) and four from Zhao et al. [13] (rs7165790 in *BLM*, rs1822744 in *TOPBP1*, rs11078671 in *RPA1* and rs401549 in *BLM*).

## 4. Discussion

We carried out an independent replication analysis of previously described SNPs associated with the risk of developing oesophagitis in NSCLC patients subject to RT. One SNP (rs7165790) from the DNA DSB repair pathway [13] and one SNP (rs4772468) from the inflammation pathway [11] were found associated with this radiation-induced adverse effect in our cohort. Although none of these variants remain significant after FDR multiple testing correction, both associations are consistent in the direction and effect size with previously published results.

López-Guerra et al. [9] reported that the CG/GG genotype of rs2868371 had a protective effect for oesophagitis grade ≥ 3 (experimental dataset: HR = 0.29, 95% CI = 0.09–0.97, *p*-value = 0.045; validation dataset: HR = 0.25, 95% CI = 0.07–0.88, *p*-value = 0.031), whereas no association with oesophagitis grade ≥ 2 was detected. The low incidence of oesophagitis grade of ≥3 in our cohort precluded the study of this phenotype, but the analysis of oesophagitis grade ≥ 2 shows an increased risk effect associated with the CG/GG genotype. This effect remains even when we replicate the analyses comparing patients by factors such as the use of concurrent chemoradiotherapy, the mean oesophageal dose and the percentage of oesophageal V40. Conflicting results have been described for the association of the SNP rs2868371 with radiation adverse effects, which have been related to variations in the distribution of the SNP genotype in different populations [25]. However, the similar distribution of the rs2868371 genotypes in our cohort (60.8% CC; 39.20% CG/GG) and the reference study (López Guerra et al. [9]: experimental dataset, 60% CC and 40% CG/GG; validation dataset, 61% CC and 39% CG/GG) rules out this possibility. The different assessments of the endpoint could also give rise to the discrepancies in the direction of the effect, as there are slight changes in the score definitions for oesophagitis from CTCAE version 3, used in López-Guerra et al. [9], and CTCAE version 4.03, used in the present study. Oesophagitis grade ≥ 3 was an unusual condition in our cohort, with a frequency below 2% compared to the frequencies of 13.6% and 9.3% described for the original study. Lastly, the nucleotide change in the SNP, a C-to-G substitution, raises the possibility of different strands genotyped for each study. Unfortunately, given that the genotyped strand is not indicated in López-Guerra et al. [9], we cannot discard this possibility.

Well aware of the limited ability to replicate radiogenomic findings, even in the larger cohorts of breast and prostate cancer [26,27,28,29], we applied a meta-analytic approach with the variants from Pu et al. [11] and Zhao et al. [13], combining our results with the previously published ones as an additional validation set. Given that an adequate a priori power for the analysis was achieved, and as our data collection was initially designed to replicate the previous studies, this technique seemed suitable to make the most out of the available information. After setting strict homogeneity requirements, our combined results preserve the statistical association in 12 out of 16 SNPs related to oesophagitis and refine their effect size measurement.

The inflammation-related variants associated with oesophagitis were rs4772468 at *FGF14* (associated with increased risk) and rs270771 at *LILRP2* (associated with decreased risk). The SNP rs7259857 in *TNFSF7* implicated in T cells’ immunity was associated with decreased risk. The SNP rs72993079 in the *TNFSF7* gene has previously been associated with an increased risk of haematuria after RT in prostate cancer patients (OR = 2.14; 95% CI = 1.60–2.87; *p*-value = 3.8 × 10^−7^) [30]. The pathophysiology behind both radiation adverse effects includes inflammation and the action of pro-inflammatory cytokines. In oesophagitis, these mechanisms are involved in mucosal inflammation and basal epithelial thinning, which can progress to denudation and ulceration [31], and in haematuria by causing damage to the urothelium, vasculature and detrusor muscle [32,33]. Besides, SNP rs940052 located in *PRKCE*, rs2707212 in *CD4*, rs7309 in *TANK* and rs1998521 in *IL15RA* also keep their association after the meta-analysis approach. All these genes are linked to the inflammatory response mediated by cytokines, which have been associated in oesophagitis with irritation or damage of the oesophageal mucosa [34].

Five genetic variants from three genes of the DNA double-strand break repair pathway maintain a significant association after the meta-analysis approach. SNPs rs7165790, rs2270132 and rs401549 in the *BLM* gene are involved in the initiation of DNA DSB repair and play an important role in the maintenance of genome stability [35]. The SNP rs1822744 is located in the *TOPBP1* gene and plays an important role in maintaining genome stability and DNA replication checkpoint control [36]. The SNP rs11078671 is located in *RPA1*, a gene proposed as a molecular matchmaker that coordinates the activities of proteins involved in strand elongation or DNA repair [37]. Recent studies suggest that acetylation of *RPA1* plays a crucial role in the repair of DNA damage via nucleotide excision repair [38]. Radiation-induced DNA damage affects the basal cells of the oesophageal epithelium, and alterations in genes involved in the repair of those mechanisms may lead to oesophageal clinical manifestations. The meta-analysis approach increases the number of replicated variants with our cohort, supporting the association of variants in genes related to inflammation and DNA DSB repair pathways.

Independent replication of findings is crucial to establish robust genetic signals in radiogenomics studies. Here, we aimed to provide an independent validation analysis for SNPs that have been previously studied in the radiogenomics context and that have been linked to the development of adverse effects following RT. Some limitations of our study could be the differences in radiation treatments; our patients were irradiated using 3D-CRT, when the referenced studies comprised different RT techniques. Moreover, our cohort had a low proportion of women. Although this is similar to the proportion of women diagnosed with lung cancer in Spain (18.47% and 18.37% in 2012 and 2015, respectively) [39], it could be related to the relatively low incidence of oesophagitis in our cohort, since previous studies have shown that the presence of oesophagitis grade ≥ 2 is almost double in women than in men [40,41,42]. Unfortunately, our modest cohort size and its characteristics did not allow to reach a desirable power for the analysis. Still, with the meta-analysis approach, most of the SNPs from inflammation- and DNA DSB repair-related genes previously associated with oesophagitis maintain their original associations. In this sense, the large dataset from the REQUITE multicentre study [43,44], supported by the Radiogenomics Consortium [45], will be available soon and will provide a unique opportunity to study those SNPs not replicated with our cohort.

In addition to clinical and dosimetry data, these variants could be included in predictive models, and after a performance evaluation, the models could be suitable for stratifying NSCLC patients before the start of RT based on their risk of developing radiation-induced oesophagitis. This personalisation of radiotherapy would allow the modification of prescription doses according to individual risk or even the consideration of alternative treatment options for patients with high risk of radiation-induced oesophagitis. Given the relatively poor prognosis of patients with locally advanced NSCLC or metastasis, it is essential to prioritise both tumour control as well as the patient’s quality of life.

## 5. Conclusions

We carried out the replication of SNPs previously associated with radiation-induced oesophagitis in an independent cohort of NSCLC patients. We managed to replicate 2 out of the 18 SNPs analysed, rs7165790 related to the DNA DBS repair pathway and rs4772468 related to inflammation.

We included our cohort as a second validation set in a meta-analysis, resulting in 12 SNPs remaining associated with radiation-induced oesophagitis.

Our findings support that genetic variants located in DSB DNA repair- and inflammation-related pathways, in conjunction with clinical and dosimetry data, could be used for NSCLC patient stratification and treatment personalisation.

## Figures and Tables

**Table 1:** Patient characteristics (*n* = 178).

Characteristics	N	%
**Sex**		
Male	152	85.39
Female	26	14.61
Age, mean (range)	63.92	(41–89)
Ethnicity, White (European)	178	100
Clinical stage		
I	5	2.81
II	10	5.62
IIIa	100	56.18
IIIb	62	34.83
Missing	1	0.56
Histology		
Squamous	90	50.56
Adenocarcinoma	74	41.57
Other	13	7.3
Missing	1	0.56
Performance status		
ECOG 0	31	17.42
ECOG 1	136	76.4
ECOG 2	9	5.06
ECOG 3	2	1.12
Smoking status		
Never	18	10.11
Ex before cancer diagnosis	77	43.26
Ex since cancer diagnosis	25	14.04
Current	56	31.46
Missing	2	1.12
No. of pack-years, mean (SD)	48.72	(34.03)
Chemotherapy		
No	15	8.43
Yes	162	91.01
Missing	1	0.56
Chemotherapy treatment modality		
Sequential	30	18.52
Concurrent	47	29.01
Induction + concurrent	79	48.77
Concurrent + consolidation	4	2.47
Induction + concurrent + consolidation	2	1.23
Radiation technique		
3D-CRT	178	100
Radiation total dose, Gy, mean (SD)	62.8	(6.94)
Radiotherapy fractionation		
Once a day, five per week	178	100
FEV1 percentage, mean (SD)	78.73	(21.1)
Missing	16	8.99
DLCO percentage, mean (SD)	77.72	(21.49)
Missing	54	30.34
Planned target volume (cm3), mean (SD)	401.94	(271.78)
Missing	8	4.49
Gross tumour volume (cm3), mean (SD)	147.85	(150.8)
Missing	55	30.9
Mean oesophageal dose, Gy, mean (SD)	22.9	(9.12)
Missing	9	5.06
Median oesophageal dose, Gy, mean (SD)	16.96	(14.62)
Missing	4	2.25
Dmax of oesophagus, mean (SD)	56.59	(12.3)
Missing	12	6.74
V40 oesophagus, %, mean (SD)	28.38	(17.04)
Missing	4	2.25
V50 oesophagus, %, mean (SD)	19.73	(15.99)
Missing	11	6.18
V60 oesophagus, %, mean (SD)	11.23	(13.75)
Missing	12	6.74
Oesophagitis		
Absence	120	67.42
CTCAE score = 1	34	19.1
CTCAE score = 2	20	11.24
CTCAE score = 3	4	2.25

Abbreviations: N: number of observations; SD: standard deviation; ECOG: Eastern Cooperative Oncology Group performance status; no. of pack-years: packs of cigarettes smoked per day/years as a smoker; 3D-CRT: three-dimensional conformal radiation therapy; FEV1: forced expiratory volume in the first second; DLCO: carbon monoxide diffusing capacity; Dmax: maximum dose; V40, V50 and V60 oesophagus: percentage of oesophagus that receives 40, 50 and 60 Gy, respectively, in terms of percentage of entire oesophagus volume; CTCAE: Common Terminology Criteria for Adverse Events (v.4.03).

**Table 2:** Validation and meta-analysis of the SNPs identified in prior studies in association with radiation adverse effects in NSCLC patients including the RADIOGEN-Lung cohort.

Sample Size	Validated SNP	Gene	Model	Discovery Set	Validation Set	RADIOGEN Cohort		Meta-Analysis	
				OR (95% CI); *p*-Value	OR (95% CI); *p*-Value	OR (95% CI); *p*-Value	*p*-adj	OR (95% CI); *p*-Value	*p*-het
534	rs1239344	*OSMR*	DOM	2.45(1.14–5.26); 0.021	4.15(1.68–10.28); 0.002	0.42(0.11–1.56); 0.198	0.396	1.80(0.59–5.54); 0.303	0.017
(201 discovery, ^ɬ^	rs7259857	*TNFSF7*	ADD	0.50(0.30–0.84); 0.008	0.50(0.28–0.90); 0.021	0.80(0.32–1.96); 0.629	0.707	**0.53(0.38–0.77); 6.23 × 10^−4^**	0.635
220 validation, ^ɬ^	rs940052	*PRKCE*	DOM	0.34(0.16–0.75); 0.007	0.37(0.15–0.90); 0.030	1.29(0.29–5.73); 0.737	0.780	**0.41(0.24–0.72); 1.75 × 10^−3^**	0.280
113 RADIOGEN)	rs4772468	*FGF14*	DOM	2.56(1.20–5.47); 0.015	2.76(1.12–6.80); 0.027	**4.36(1.15–16.46); 0.029**	0.179	**2.86(1.68–4.87); 1.07 × 10^−4^**	0.788
	rs2707212	*CD4*	DOM	2.70(1.23–5.92); 0.013	2.23(1.04–4.79); 0.040	1.45(0.40–5.27): 0.569	0.707	**2.26(1.37–3.74); 1.52 × 10^−3^**	0.722
	rs270771	*LILRP2*	DOM	0.28(0.08–0.93); 0.037	0.10(0.02–0.66); 0.017	0.02(0.00–1.20); 0.062	0.236	**0.17(0.07–0.46); 4.41 × 10^−4^**	0.380
	rs1998521	*IL15RA*	ADD	1.78(1.03–3.08); 0.037	1.82(1.02–3.26); 0.043	0.75(0.28–1.93); 0.550	0.707	**1.57(1.09–2.28); 0.015**	0.247
	rs7309	*TANK*	DOM	2.42(1.06–5.55); 0.036	3.02(1.04–8.77); 0.043	1.61(0.34–7.43); 0.543	0.707	**2.43(1.34–4.45); 3.67 × 10^−3^**	0.803
533	rs7165790	*BLM*	ADD	0.59(0.37–0.97); 0.037	0.45(0.22–0.94); 0.032	**0.16(0.04–0.65); 0.010**	0.098	**0.49(0.34–0.73); 3.58 × 10^−4^**	0.223
(250 discovery, ^§^	rs2270132	*BLM*	DOM	2.59(1.27–5.26); 0.009	1.75(0.65–4.74); 0.268	0.90(0.25–3.22); 0.871	0.870	**1.93(1.14–3.28); 0.013**	0.355
170 validation, ^§^	rs4873772	*PRKDC*	REC	7.17(1.77–29.05); 0.006	1.28(0.30–5.46); 0.743	0.53(0.05–5.34); 0.590	0.707	2.00(0.46–8.80); 0.355	0.094
113 RADIOGEN)	rs1822744	*TOPBP1*	ADD	1.86(1.10–3.13); 0.021	1.24(0.69–2.24); 0.473	1.81(0.77–4.24); 0.170	0.396	**1.59(1.12–2.28); 9.71 × 10^−3^**	0.570
	rs11078671	*RPA1*	REC	4.17(1.19–14.61); 0.026	1.69(0.50–5.71); 0.400	3.82(0.87–16.71); 0.075	0.236	**2.88(1.36–6.13); 5.69 × 10^−3^**	0.545
	rs401549	*BLM*	ADD	1.91(1.14–3.20); 0.013	1.08(0.54–2.17); 0.821	1.94(0.73–5.08); 0.179	0.396	**1.61(1.10–2.36); 0.013**	0.400
	rs1776139	*EXO1*	DOM	0.45(0.21–0.98); 0.044	0.69(0.28–1.68); 0.414	8.03(0.78–82.15); 0.079	0.236	0.83(0.29–2.42); 0.738	0.067
	rs10514249	*XCRR4*	REC	0.39(0.17–0.89); 0.024	0.86(0.27–2.69); 0.792	1.58(0.37–6.63); 0.532	0.707	0.62(0.34–1.15); 0.131	0.207
				**HR (95% CI); *p*-Value**	**HR (95% CI); *p*-Value**	**HR (95% CI); *p*-Value**	***p*-adj**		
463	rs2868371	*HSPB1*	REC	0.29(0.09–0.97); 0.045	0.25(0.07–0.88); 0.031	**3.72(1.49–9.25); 0.004**	0.083		
(120 discovery, ^҂^									
181 validation, ^҂^									
162 RADIOGEN)									
360	rs1800469	*TGFB1*	DOM	2.47(1.02–6.02); 0.045	2.53(1.14–5.63); 0.023	0.67(0.28–1.63); 0.385	0.694		
(97 discovery, ^Ø^									
101 validation, ^Ø^									
162 RADIOGEN)									

Abbreviations: SNP: single-nucleotide polymorphism; NSCLC, non-small-cell lung cancer; DOM, dominant; REC, recessive; ADD, additive; OR, odds ratio; CI, confidence interval; *p*-adj; FDR-adjusted *p*-value; *p*-het, Cochran Q statistic heterogeneity test *p*-value; HR, hazard ratio. ^ɬ^ Pu et al. (2014) [11]: adjusted for age, sex, pack year, clinical stage, performance status, concurrent chemoradiotherapy, FEV1 percentage, DLCO percentage, PTV volume and median oesophagus dose. ^§^ Zhao et al. (2016) [13]: adjusted for age, sex, pack-year, clinical stage, performance status, concurrent chemoradiotherapy, FEV1 percentage, DLCO percentage, PTV volume and mean oesophagus dose. ^҂^ López-Guerra et al. (2011) [9]: adjusted for age, Karnofsky performance status (KPS), disease stage, radiotherapy fractionation, smoking status, mean oesophagus dose and oesophagus V40%. ^Ø^ López-Guerra et al. (2012) [10]: adjusted for age, smoking status, Karnofsky performance status (KPS), tumour stage, concurrent chemotherapy and radiation total dose. In bold OR and *p*-Value of replicated SNPs.

## Data Availability

The data presented in this study are available on request from the corresponding author. The data are not publicly available due to the consent provided by participants did not include the consent to make their data publicly available.

## Supplementary Materials

The following are available online at https://www.mdpi.com/2072-6694/13/6/1447/s1: Figure S1: SNPrs3868371 in HSPB1 associated with oesophagitis CTCAE grade ≥ 2; Figure S2: Freedom from radiation oesophagitis CTCAE grade ≥ 2 as a function of time from the start of RT by SNP rs1800469 genotypes in TGFB1; Table S1: Studies of SNPs associated with adverse effects after radiotherapy in non-small-cell lung cancer patients with a validation cohort; Table S2: Description of variables included in the replication analysis.

## Abbreviations

RT: radiation therapy; NSCLC: non-small-cell lung cancer; DSB: double-stand breaks; CTCAE: Common Terminology Criteria for Adverse Events; ECOG: Eastern Cooperative Oncology Group; 3D-CRT: three-dimensional conformal radiation therapy; CT: computed tomography; GTV: gross tumour volume; FDG-PET: fluorodeoxyglucose–positron emission tomography; CTV: clinical target volume; PTV: planning tumour volume; FOCAL: fusion of CT and linear accelerator unit; ICRU: International Commission of Radiation Units and Measurements; DVH: dose–volume histogram; OARs: organs at risk; OR: odds ratio; HR: hazard ratio; CI: confidence interval.
