# Supplementary material for: Validation of Polymorphisms Associated with the Risk of Radiation-Induced Oesophagitis in an Independent Cohort of Non-Small-Cell Lung Cancer Patients
Source: Cancers (Basel). 2021 Mar 22;13(6):1447. doi: 10.3390/cancers13061447 (PMC8004670; doi:10.3390/cancers13061447)
Supplement: Supplementary file 1 [file cancers-13-01447-s001.pdf]

cancers

MDPI

Supplementary Materials: Validation of Polymorphisms Associated with the Risk of Radiation-Induced Esophagitis in an Independent Cohort of Non-Small Cell Lung Cancer Patients

Miguel E. Aguado-Barrera, Laura Martínez-Calvo, Juan Fernández-Tajes, Patricia Calvo-Crespo, Begoña Taboada-Valladares, Ramón Lobato-Busto, Antonio Gómez-Caamaño, and Ana Vega

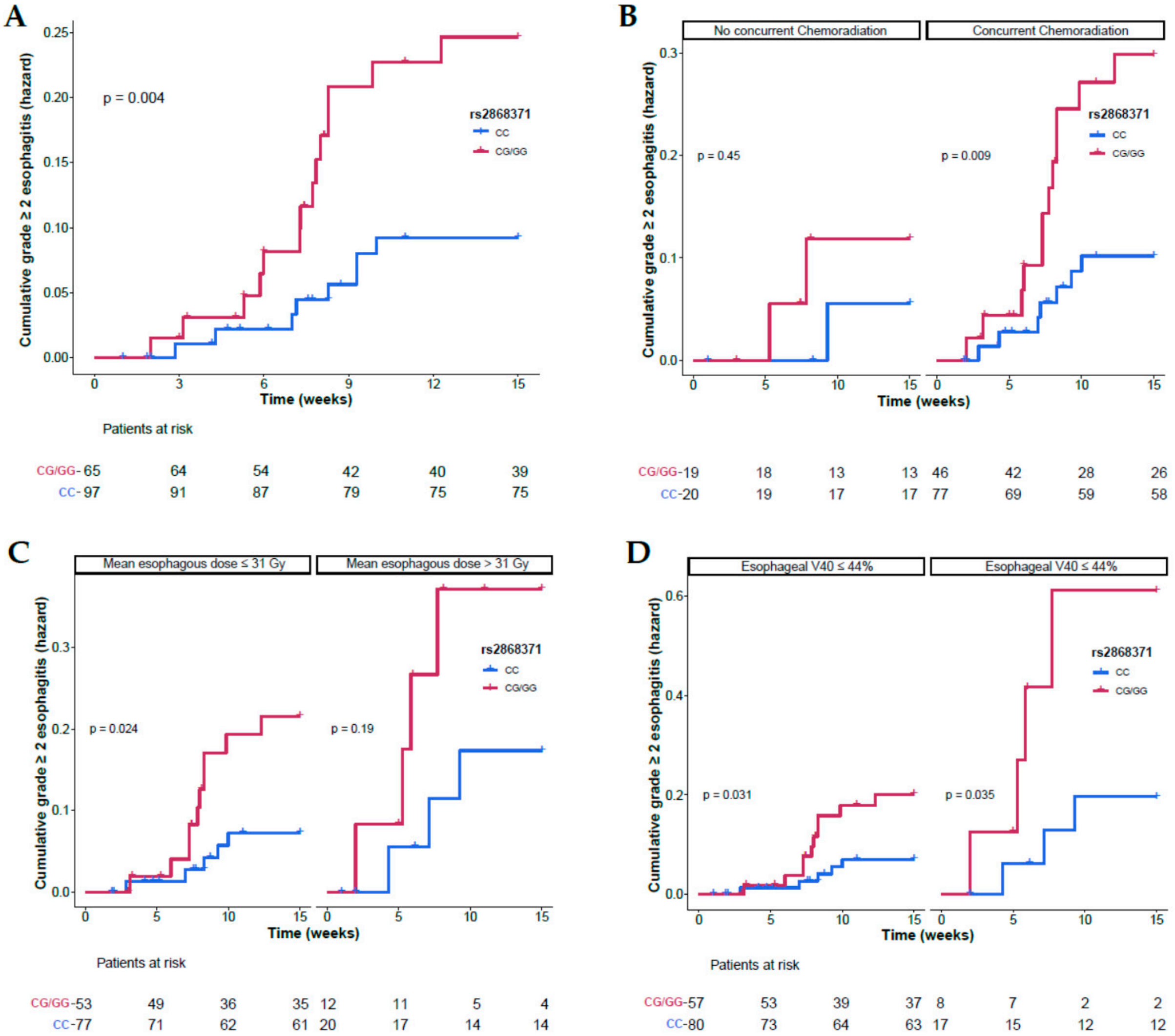

Figure S1. SNPrs3868371 at HSPB1 associated with associated with esophagitis CTCAE grade ≥ 2. (**A**) Kaplan-Meier curve for esophagitis grade ≥ 2 as a function of time in non-small-cell lung cancer patients for *HSPB1* rs2868371 genotypes. Cumulative probability of esophagitis grade ≥ 2 as a function of time in non-small-cell lung cancer patients according to *HSPB1* rs2868371 genotypes comparing with the use of concurrent chemoradiation **(B)**, dosimetric parameters: mean esophagus dose ≤31 Gy vs >31 Gy **(C)** and esophagus V40 ≤44% vs >44% **(D)**. The plots were generated using survminer [1] and gridExtra [2] R packages.

cancers

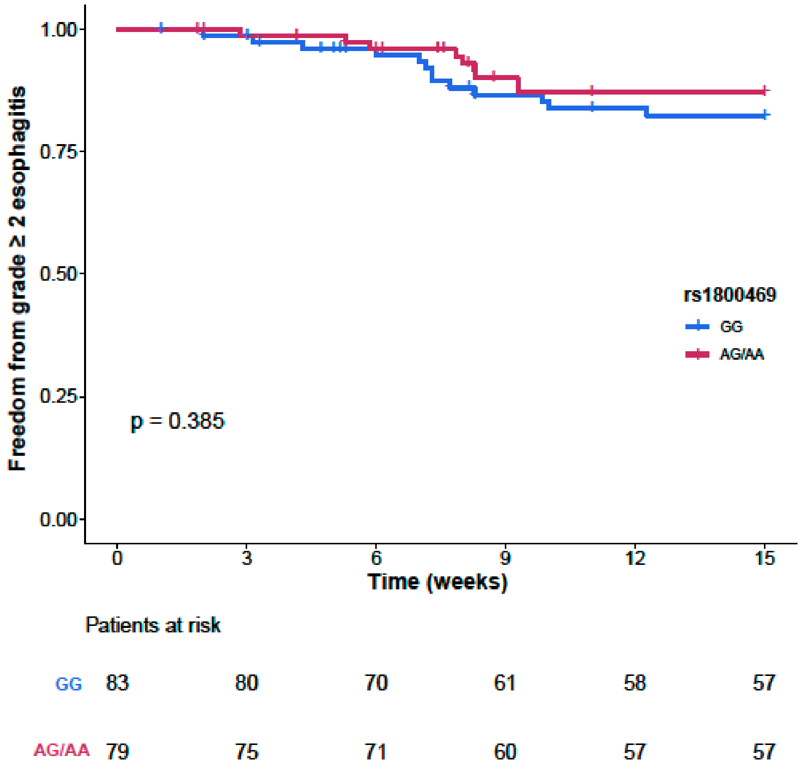

Figure S2: Freedom from radiation esophagitis CTCAE grade ≥ 2 as a function of time from the start of RT by Scheme 1800469. genotypes at TGFB1. Freedom from radiation esophagitis CTCAE grade ≥ 2 as a function of time from the start of RT by SNP rs1800469 genotypes at TGFB1. The plot was generated using the survminer R package [1].

cancers MDPI

Table S1. Studies of SNPs associated with radiation-induced toxicity in non-small-cell lung cancer patients with a validation cohort.

First author, year [Ref]	Adverse Effect *	Sample size	Type of Radiotherapy (%)	Treatment modality (%)	Clinical Stage	Pathway-related genes	Validated SNP	Gene	Model	MAF
López-Guerra (2011) [3]	Esophagitis (grade = 3)#	301(120 discovery, 181 replication)	3D (55), IMRT (38), Proton (7)	RT(20), CRT(80)	NSCLC (I-IV)	Heat-shock protein	rs2868371	*HSPB1*	NA	NA
López-Guerra (2012) [4]	Esophagitis (grade = 3)#	198(97 discovery, 101 replication)	3D (43.3), IMRT (56.7)	C+R(8.1), C+CRT (34), CRT(50.5), RT(7.2)	NSCLC (II-IV)	Inflammatory processes	rs1800469	*TGFB1*	NA	NA
Pu (2014) [5]	Esophagitis (grade ≥ 2)	421 (201 discovery, 220 validation)	2D (12), 3D (30), IMRT (55), Proton (18)	RT (42), CRT(58)	NSCLC (I-IIIB)	Inflammation-related genes	rs1239344	*OSMR*	DOM	0.449
							rs7259857	*TNFSF7*	ADD	0.318
							rs940052	*PRKCE*	DOM	0.495
							rs4772468	*FGF14*	DOM	0.253
							rs3819721 ≠	*TAP1*	DOM	0.285
							rs2707212	*CD4*	DOM	0.291
							rs270771	*LILRP4*	DOM	0.013
							rs1998521	*IL15RA*	ADD	0.423
							rs7309	*TANK*	DOM	0.408
							rs204993 ≠	*AGER*	ADD	0.301
Zhao (2016) [6]	Esophagitis (grade ≥ 2)	420 (250 discovery, 170 validation)	2D (30), 3D(42), IMRT (23),Proton (5)	RT (26), CRT(74)	NSCLC (I-IIIB)	DNA DSB repair pathway	rs7165790	*BLM*	ADD	0.434
							rs8176257	*BRCA1*	REC	0.290
							rs2270132	*BLM*	DOM	0.391
							rs12516	*BRCA1*	REC	0.342
							rs1799966	*BRCA1*	REC	0.355
							rs4873772	*PRKDC*	REC	0.308
							rs1822744	*TOPBP1*	ADD	0.430
							rs11078671	*RPA1*	REC	0.449
							rs401549	*BLM*	ADD	0.365
							rs1776139	*EXO1*	DOM	0.482
							rs10514249	*XCRR4*	REC	0.406

Abbreviations: NA: Not available; SNP: single nucleotide polymorphism; 3D: three dimensional conformal radiotherapy; IMRT: intensity-modulated radiotherapy; 2D: two-dimensional radiotherapy; RT: radiotherapy; C: chemotherapy; CRT: chemoradiotherapy; NSCLC: Non-small cell lung cancer; SNP: single nucleotide polymorphism; MAF: minor allele frequency; HR: Hazzard Ratio; OR: Odds Ratio. * Adverse effects grades scored according to the National Cancer Institute Common Terminology Criteria for Adverse Events (CTCAE). ≠ SNPs could not be included in the analyses for genotyping design reasons. # Due to the low incidence of esophagitis, we considered grade ≥ 2 in our cohort.

Table S2. Description of variables included in the replication analysis.

Covariates	Studies: López-Guerra (2011) ≠ [3]	López-Guerra (2012) ≠ [4]	Pu (2014) [5]	Zhao (2016) [6]
**Sex**	Male<br>Female	Male<br>Female	Male<br>Female	Male<br>Female
**Age**	≤ 65<br>> 65	≤ 63<br>> 63	n (years)	n (years)
**Ethnicity**	White (European)<br>Other	White (European)<br>Other	-	-
**Stage**	I, II<br>III, IV	I, II, IIIA<br>IIIB, IV	I<br>II<br>IIIA<br>IIIB	I<br>II<br>IIIA<br>IIIB
**Histology**	Squamous cell<br>Adenocarcinoma<br>NSCLC, NOS	Squamous cell<br>Adenocarcinoma<br>NSCLC, NOS	-	Squamous cell<br>Adenocarcinoma<br>Large Cell<br>Others
**Performance Status**	KPS ≥ 80 †<br>KPS < 80 ø	KPS ≥ 80 †<br>KPS < 80 ø	ECOG = 0<br>ECOG = 1<br>ECOG = 2–4	ECOG = 0<br>ECOG = 1<br>ECOG = 2–4
**Smoking status**	Former/never<br>Current	Never<br>Former<br>Current	-	-
**No. of pack years**	≤ 45<br>> 45	≤ 44<br>> 44	n (packs/year)	n (packs/year)
**Concurrent chemoradiation**	No<br>Yes	No<br>Yes	No<br>Yes	No<br>Yes
**Radiation technique**	3D<br>IMRT<br>Proton	3D<br>IMRT	3D<br>2D<br>IMRT<br>Proton	3D<br>2D<br>IMRT<br>Proton
**Radiation total dose**	≤ 63<br>>63	≤ 69<br>>69	-	-
**Radiotherapy fractionation**	once a day<br>twice a day	once a day<br>twice a day	-	-
**FEV1 percentage**	-	-	(%)	(%)
**DLCO percentage**	-	-	(%)	(%)
**PTV**	-	-	cm^3	cm^3
**Mean esophageal dose**	≤ 31<br>> 31	≤ 33<br>> 33	-	Gy
**Median esophageal dose**	-	-	Gy	-
**Dmax of esophagus**	≤ 68<br>> 68	≤ 69<br>> 69	-	-
**V40% esophagus**	≤ 44<br>> 44	-	-	-
**V50% esophagus**	≤ 38<br>>38	≤ 41<br>> 41	-	-
**V60% esophagus**	-	≤ 29<br>>29	-	-
**Esophagitis ***	grade <2 vs ≥ 2 &<br>grade <3 vs ≥ 3	grade <2 vs ≥ 2 &<br>grade <3 vs ≥ 3	grade <2 vs ≥ 2	grade <2 vs ≥ 2

≠ Variables were analysed as categorical into the groups described in each covariate. † Equivalence KPS ≥ 80 = ECOG ≤ 1. Ø Equivalence KPS < 80 = ECOG ≥ 2. * Adverse effects grades scored according to the National Cancer Institute Common Terminology Criteria for Adverse Events (CTCAE). - Variable not considered in the study. Abbreviations: NSCLC: non-small cell lung cancer; NOS: non-small cell lung carcinoma, not otherwise specified; KPS: Karnofsky performance score; ECOG: Eastern Cooperative Oncology Group performance status; No. of pack years = (packs of cigarettes smoked per day)/(years as a smoker); 3D: three dimensional conformal radiotherapy; 2D: two-dimensional radiotherapy; IMRT: intensity-modulated radiotherapy; FEV1: forced expiratory volume in the first second; DLCO: carbon monoxide diffusing; PTV:

cancers MDPI

planning tumor volume; Dmax: maximum dose; V40, V50 and V60 oesophagus: percentage of oesophagus that receives 40, 50 and 60 Gy respectively in terms of percentage of the entire oesophagus volume..

Supplementary References

1. Kassambara, A.; Kosinski, M.; Biecek, P.; Fabian, S. **2020**. survminer: Drawing Survival Curves using “ggplot2” (version 0.4.8). R Package. URL https://cran.r-project.org/web/packages/survminer/index.html/ (accessed on 1st March 2021).
2. Auguie, B. **2017**. gridExtra: Miscellaneous Functions for “Grid” Graphics (version 2.3). R Package. URL https://cran.r-project.org/web/packages/gridExtra/index.html/ (accessed on 1st March 2021)
3. Lopez Guerra, J.L.; Wei, Q.; Yuan, X.; Gomez, D.; Liu, Z.; Zhuang, Y.; Yin, M.; Li, M.; Wang, L.E.; Cox, J.D.; et al. Functional promoter rs2868371 variant of HSPB1 associates with radiation-induced esophageal toxicity in patients with non-small-cell lung cancer treated with radio(chemo)therapy. *Radiother. Oncol.* **2011**, doi:10.1016/j.radonc.2011.08.039.
4. Guerra, J.L.L.; Gomez, D.; Wei, Q.; Liu, Z.; Wang, L.E.; Yuan, X.; Zhuang, Y.; Komaki, R.; Liao, Z. Association between single nucleotide polymorphisms of the transforming growth factor β1 gene and the risk of severe radiation esophagitis in patients with lung cancer. *Radiother. Oncol.* **2012**, doi:10.1016/j.radonc.2012.08.014.
5. Pu, X.; Wang, L.; Chang, J.Y.; Hildebrandt, M.A.T.; Ye, Y.; Lu, C.; Skinner, H.D.; Niu, N.; Jenkins, G.D.; Komaki, R.; et al. Inflammation-related genetic variants predict toxicity following definitive radiotherapy for lung cancer. *Clin. Pharmacol. Ther.* **2014**, doi:10.1038/clpt.2014.154.
6. Zhao, L.; Pu, X.; Ye, Y.; Lu, C.; Chang, J.Y.; Wu, X. Association between genetic variants in DNA double-strand break repair pathways and risk of radiation therapy-induced pneumonitis and esophagitis in non-small cell lung cancer. *Cancers (Basel).* **2016**, doi:10.3390/cancers8020023.
